# Description and optimization of a multiplex bead-based flow cytometry method (MBFCM) to characterize extracellular vesicles in serum samples from patients with hematological malignancies

**DOI:** 10.1038/s41417-022-00466-1

**Published:** 2022-04-27

**Authors:** Lin Li, André Görgens, Veronika Mussack, Elena Pepeldjiyska, Anne Sophie Hartz, Andreas Rank, Jörg Schmohl, Doris Krämer, Samir El Andaloussi, Michael W. Pfaffl, Helga Schmetzer

**Affiliations:** 1 Working-group: Immune-Modulation, Medical Department III, University Hospital of Munich, Munich, Germany; 2 Department of Laboratory Medicine, Division of Biomolecular and Cellular Medicine, Karolinska Institutet, Stockholm, Sweden; 3 Department of Animal Physiology and Immunology, TUM School of Life Sciences Weihenstephan, Technical University of Munich, Freising, Germany; 4 Department of Hematology and Oncology, University Hospital of Augsburg, Augsburg, Germany; 5 Department of Hematology and Oncology, Hospital of Stuttgart, Stuttgart, Germany; 6 Department of Heamatology, Oncology and Palliative Care, Ameos Klinikum Mitte, Bremerhaven, Germany

**Keywords:** Biomarkers, Immunization, Targeted therapies, Cell biology

## Abstract

Extracellular Vesicles (EVs) are membranous vesicles produced by all cells under physiological and pathological conditions. In hematological malignancies, tumor-derived EVs might reprogram the bone marrow environment, suppress antileukemic immunity, mediate drug resistance and interfere with immunotherapies. EVs collected from the serum of leukemic samples might correlate with disease stage, drug-/immunological resistance, or might correlate with antileukemic immunity/immune response. Special EV surface protein patterns in serum have the potential as noninvasive biomarker candidates to distinguish several disease-related patterns ex vivo or in vivo. EVs were isolated from the serum of acute myeloid leukemia (AML), acute lymphoid leukemia (ALL), chronic lymphoid leukemia (CLL) patients, and healthy volunteers. EVs were characterized by transmission electron microscopy and fluorescence nanoparticle tracking analysis, and EV surface protein profiles were analyzed by multiplex bead-based flow cytometry to identify tumor- or immune system-related EVs of AML, ALL, CLL, and healthy samples. Aiming to provide proof-of-concept evidence and methodology for the potential role of serum-derived EVs as biomarkers in leukemic versus healthy samples in this study, we hope to pave the way for future detection of promising biomarkers for imminent disease progression and the identification of potential targets to be used in a therapeutic strategy.

## Introduction

### Leukemia

Leukemia and lymphoma are blood malignancies that affect people of all ages and result in approximately 23,000 deaths in the United States per year [1]. Acute myeloid (AML) [2], lymphoid (ALL) [3], or chronic lymphoid leukemia (CLL) [4] are clonal diseases with uncontrolled proliferation of myeloid or lymphoid leukemic cells, that can be identified and characterized by flow cytometry. Rates of complete remission (CR), prognosis and survival depend on the grade of anemia, thrombocytopenia, white blood cell expansion and karyotypes. Risk-adapted therapies for AML, ALL, and CLL patients consist of chemotherapy with/without stem cell transplantation (SCT), but the rate of early failures and relapses is still unsatisfying. Since relapse rates in successfully treated AML, ALL, and CLL patients are high, new therapy options are needed [5, 6].

### Immune surveillance

Effective immune surveillance of patients with hematologic malignancies such as leukemia is mediated by cellular and noncellular arms of the innate and adaptive immune system. The innate immune system includes macrophages, dendritic cells (DC), and natural killer (NK) cells, which respond quickly to an immunological threat. The adaptive immune system includes T and B cells, which mediate tumor immunity by antigen-specific responses and provide long-lasting protection by effector-memory responses [6, 7]. Furthermore, other cells at the interface of the innate and the adaptive immune system (e.g cytokine-induced killer cells (CIK) or invariant natural killer T-cells (iNKT)) are important mediators in antitumor-, autoimmune-, and antimicrobial responses and tumor surveillance. Moreover, soluble key players and mediators of immune reactions trafficking through the body like hormones, (B-cell-derived) antibodies, cytokines, chemokines, and several macromolecules, membranous vesicular entities, such as extracellular vesicles (EVs), circulating nucleic acids and their derivatives appear equally relevant in immunomodulatory mechanisms [6–9].

### Tumor and immune monitoring

Regularly, anergy of T cells or other immune cells can be regularly reverted to anti-leukemic functionality. It is important to understand leukemia-related as well as antileukemic processes. Therefore analyzing and monitoring the involvement of different (activating or inhibitory) cells, soluble or even the smallest molecules in antileukemic processes is necessary: qualitative and quantitative flow cytometric evaluations and monitoring of leukemic- and immune-reactive cells and their subtypes in a tumor-, inflammatory or infectious immunological context (in vivo or in various cell cultures) is important and informative to evaluate and monitor inhibitory or activating (antigen specific) cell populations [6, 8, 9].

In recent years, previously unrecognized influences of physical factors such as physiological hypoxia and other molecules (e.g. soluble molecules or circulating vesicles (EVs)) have been put to the test, and their role in tumor or immune activation or monitoring of various events could contribute to further understanding of such processes [10].

### Extracellular vesicles (EVs)

Extracellular vesicles (EVs) are membranous vesicles produced by all cells under physiological and pathological conditions [11]. EV mediated information transfer allows a crosstalk between cells of the hematopoietic system and interactions between hematopoietic cells and local or distant tissue cells [12]. Emerging evidence suggests that EVs play a key role in the regulation of the entire physiology, including tissue differentiation and repair, hematopoietic stem cell development, coagulation, pregnancy or immune surveillance [13–15]. Due to the heterogeneity, the small size of EVs and the lack of standardization and, in particular, a qualified method to analyze multiple parameters of single EVs, a qualitative and quantitative detection and evaluation of EVs is challenging. As of now, only a few EV surface markers have been reliably linked to specific cell sources [7, 16].

### Methods to isolate and characterize EVs

EVs can be isolated by various methods, e.g. ultracentrifugation, precipitation, size exclusion chromatography, ultrafiltration, and other immunoaffinity-based binding strategies [17]. A combination of isolation methods will increase the EV purity. Isolated pure EVs can be characterized by multiple methods to prove their specific properties. For example, EV morphology and size can be assessed by transmission electron microscopy (TEM) [18]. Moreover, (fluorescence) nanoparticle tracking analysis (fNTA) [15] allows to determine particle size and concentration of vesicle preparations. EV specific (surface) protein markers (e.g. CD9, CD63, CD81) can be detected using Western blot and different flow cytometry-based methods.

While high sensitivity and high-resolution methods like Imaging Flow Cytometry (IFCM) are very promising to unravel EV heterogeneity and to quantify subsets more accurately, such methods also require further benchmarking against other methods and standardization before they can be used widely and ultimately in a clinical context [15, 19]. Here, we instead have further explored the use of multiplex bead-based flow cytometry (MBFCM) for analysis of EVs in human serum samples. MBFCM does not provide information about single EVs, however, it is a robust method to assess the overall EV surface protein signature in isolated EV samples including human body fluids such as blood serum/plasma [14, 15]. We previously have optimized this multiplex bead-based method with a focus on cell culture-derived EVs [20], and here we aim to further explore MBFCM to characterize EVs from patients suffering from various hematological malignancies and relate results to clinical data in a first proof-of-concept study.

The aim of this study was to (1) prepare EVs from standard serum samples of leukemia patients and healthy donors; (2) characterize resulting EV preparations by standard methods, i.e., TEM and fNTA; (3) evaluate the use of MBFCM for comparing the overall EV surface protein composition on EVs in minimally processed samples from leukemia patients versus healthy donors; (4) evaluate resulting data for potential correlation with patient’s cellular or clinical data. (5) Proof of concept deduction of EV-associated prognostic and diagnostic classification strategy for leukemia patients.

## Materials and methods

### Patients’ characteristics and diagnostics

Samples from patients with AML (*n* = 4), ALL (*n* = 3), CLL (*n* = 2), and healthy donors (*n* = 4), provided by the University Hospitals of Munich, Stuttgart, Oldenburg, and Augsburg, were collected after obtaining patients’ written informed consent in accordance with the Helsinki protocol and the local Ethic Committee (Pettenkoferstr. 8a, 80336 Munich, Ludwigs-Maximilians-University Hospital in Munich; Vote-No 339-05). The mean age of AML patients was 60.75 (range: 38–81) years, of ALL patients 59 (range: 57–62) years, of CLL patients 80 (range: 76–84) years and of healthy controls 36 (range: 29–56) years (Table 1).

**Table 1:** Characteristics of WB-samples from healthy donors and AML, ALL, CLL-patients.

	Pat.Nr	Age/Sex	Dgn. subtypes	stage	ML LC (%)	IC LC (%)	blast phenotype (CD)	Risk Stratification	WBC [G/I]	Hb [g/dl]	PLT [G/L]	Response to (induction)-chemotherapy
**AML**	1562	38/M	p/nd	Dgn	12	23	**34**,**117**,13,56, HLA-DR	Adverse^1^	1.79	9.9	190	CR
	1564	68/M	p/M4	Rel.	72	70	**34**,**117**,13,33, 65,56, HLA-DR	Adverse^1^	21.7	8.8	65	NCR
	1574	56/M	s/nd	Dgn	77	60	**34**, **117**,15,19, HLA-DR	Adverse^1^	4.5	9.4	58	NCR
	1584	81/M	s/nd	Dgn	40	82	**15**,**65**,**56**,33, HLA-DR	Adverse^1^	4.92	8.9	77	NCR
**ALL**	1587	58/M	c/B-ALL	Dgn	34	40	**34**,**19**,15,10,56, HLA-DR	High^2^	4.83	8.6	478	NCR
	1588	62/M	c/B-ALL	Dgn	1	18	**34**,**19**,56,20, HLA-DR	Standard^2^	1.74	11.9	186	NCR
	1605	57/F	c/B-ALL	Dgn	80	78	**34**,**19**,20,22,10,56, HLA-DR	Highest^2^	133	12.1	76	NCR
**CLL**	1589	84/M	p/B-CLL	Dgn	30	71	**5**,**19**,20,15,23,56, HLA-DR	A^3^	10.15	15.3	142	nd
	1591	76/F	p/B-CLL	Dgn	52	30	**5**,**19**,15,20, HLA-DR	A^3^	23.46	13.5	203	nd
**H**	1561	30/F	nd	nd	nd	nd	nd	nd	nd	nd	nd	nd
	1566	29/F	nd	nd	nd	nd	nd	nd	nd	nd	nd	nd
	1576	56/M	nd	nd	nd	nd	nd	nd	nd	nd	nd	nd
	1582	29/M	nd	nd	nd	nd	nd	nd	nd	nd	nd	nd

*AML* acute myeloid leukemia; ALL acute lymphoid leukemia; CLL chronic lymphoid leukemia; H healthy donors; Pat. Nr. Patient’s number; F female; M male; p primary; s secondary; c: common; CD Cluster of differentiation; dgn first diagnosis; rel relapse; CR Complete remission; NCR no complete remission; pers. persisting disease; PLT platelets; WBC white blood cells; IC LC immune cytologically detected leukemic cells; ML LC morphologically detected leukemic cells in peripheral blood; nd no data.

^1^AML patients were prognostically classified based on the National Comprehensive Cancer Network (NCCN) guidelines as “favorable”, “intermediate” or “adverse risk”.

^2^Risk stratification for adult ALL was based on the Study Group for Adult Acute Lymphoblastic Leukemia (GMALL) as “standard”, “high” or “highest risk”.

^3^According to Binet-classification, CLL patients were classified by Binet A, Binet B, Binet C. Bold blast markers were used for (co)expression analyses.

AML patients presented with primary (p) (*n* = 2) or secondary (s) AML (*n* = 2) disease, three patients were analyzed at first diagnosis, one at relapse. According to National Comprehensive Cancer Network (NCCN) guideline, all four AML patients were risk categorized as Adverse. All three ALL patients were classified as c-B/ALL according to the European Group of Immunophenotyping of Leukemias classification and were risk-categorized as “standard” (*n* = 1), “high” (*n* = 1) or “highest risk” (*n* = 1) based on the Study Group for Adult Acute Lymphoblastic Leukemia (GMALL). The two CLL patients were classified as p/B-CLL and risk-categorized as Binet A (Table 1). Leukemia samples contained between 18 and 82% of immune cytologically detected leukemic cells (IC leukemic cells) and decreased sequence of monocytes, T-, B-, and NK-cells compared to healthy samples (Table 2).

**Table 2:** Cellular composition of AML, ALL, CLL, and healthy samples.

	Pat.Nr	CD14+ expressing cell	CD19+ expressing cell	CD3+ expressing cell	CD56+ expressing cell	CD56+/CD3- expressing cell
**AML**	P1562	0.12	2.88	64.96	9.52*	2.96*
	P1564	0.25	3.17	10.27	5.24*	3.34*
	P1574	3.87	39.5*	11.93	1.02	0.32
	P1584	8.15	0.77	8.55	26.9*	17.35*
**ALL**	P1587	9.53	35.04*	32.87	31.72*	21.17*
	P1588	1.43	14*	32.73	5.08*	3.82*
	P1605	5.75	68*	10.78	8.23*	5.52*
**CLL**	P1589	5.03	71*	11.38	9.4*	4.42*
	P1591	1.08	45.92*	11.9	1.78	1.77
**H**	1561	5.46	2.39	20.75	9.92	6.67
	1566	3.15	0.74	11.83	4.67	3.16
	1576	9.54	2.14	8.96	12.28	4.78
	1582	1.59	0.65	nd	5.16	2.83

* (aberrant) expression of these markers on leukemic cells.

### Preparation of serum samples

Around 10 ml serum were taken from patients with AML, ALL, CLL, and healthy donors. Cells were sedimented and serum retained by centrifugation at room temperature for 10 min at 2000x *g*. The resulting supernatants (containing EVs) were aliquoted in 0.5 ml tubes and stored at -80 °C until further processing.

### Enrichment of EVs from serum samples by immunoaffinity

As recommended by MISEV2018 guidelines, EVs were characterized by TEM and fNTA [20]. For this purpose, EVs were enriched from 1.5 ml serum, respectively, by immunoaffinity applying the Exosome isolation kit pan, human (Miltenyi Biotec, Germany) as recommended by the manufacturer including a one-by-one dilution with 1x PBS prior to an additional centrifugation at 10,000x *g* for 45 min. After elution in 100 µl isolation buffer, EV preparations were vacuum evaporated to a final volume of around 20 µl, recording the exact volumes for later re-calculations.

### Transmission electron microscopy (TEM)

TEM was performed to evaluate EV morphology and size, and to assess the purity of enriched EV fractions. Therefore, five μl of freshly isolated EV preparations were loaded onto formvar carbon-coated grids (Nickel Grid 200 mesh; Electron Microscopy Sciences, USA) and left there to adhere for five minutes prior to five minutes of negative staining with 2% aqueous uranyl acetate at room temperature in the dark. Surplus liquids were removed. Images were acquired of air-dried grids on the same day at 80 kV using the Zeiss EM 900 instrument (Zeiss, Germany) equipped with a wide-angle dual-speed 2KCCD camera.

### Fluorescence Nanoparticle Tracking Analysis (fNTA)

Particle diameter/size distribution and concentration in resulting EV preparations were analyzed by fNTA. For discrimination between biological and non-biological particles a fluorescent membrane dye was used. Analyses were performed on a ZetaView PMX110 instrument (Particle Metrix, Germany), and the corresponding software version 8.05.12 SP1 was used as described before [21]. In brief, EV preparations were stained with 5 µg/ml CellMask Orange Plasma Membrane Stain (Invitrogen, USA) and incubated for 30 minutes at 37 °C prior to appropriate sample dilution in 1x PBS. After instrument calibration, the temperature was clamped at 23 °C, and the pre-acquisition parameters to measure in the fluorescence mode were set to a shutter of 70, frame rate of 30 and a sensitivity of 95 % at high resolution. Post-acquisition parameters were as follows: minimum brightness of 25, size range of 5–1000 nm and a trace length of 15. Two cycles of measurement at eleven positions were conducted. To obtain EV concentrations of initial serum samples, a recalculation according to Eitan et al. [22] was applied accounting for sample dilution and EV sample and serum volume. Additionally, thereby obtained EV concentrations were normalized to the number of one million white blood cells.

### Multiplex Bead-Based Flow Cytometry (MBFCM)

Serum samples were subjected to multiplex bead-based EV flow cytometry analysis (MBFCM; MACSPlex Exosome Kit, human, Miltenyi Biotec) as described previously [15]. In brief, EV-containing serum samples were thawed and (without further purification) subjected to centrifugation at 2,500 x g for 15 minutes before supernatants were processed as follows: Unless indicated otherwise, 30 µL of sample was diluted 1:1 with MACSPlex buffer (MPB) to a total volume of 60 µL and loaded onto wells of a pre-wet and drained MACSPlex 96-well 0.22 μm filter plate before 8 μL of MACSPlex Exosome Capture Beads (containing 39 different antibody-coated bead subsets) were added to each well and counterstained with APC-labelled pan-tetraspanin antibodies (CD9, CD63, CD81) as described previously. Unless mentioned otherwise in the results section data was analyzed as described before [15].

### Statistical analysis

Data is presented as mean ± standard deviation unless otherwise stated. For the comparison of two groups a paired t-test was used, more than two groups were analyzed applying one-way analysis of variance (ANOVA) with Benjamini-Hochberg adjustments for multiple comparisons [23]. Differences were considered as ‘not significant’ in cases with p-values > 0.1, as ‘borderline significant’ (^#^) with p-values between 0.05 and 0.1, as ‘significant’ (*) with *p* values between 0.01 and 0.05, as ‘highly significant’ (**) with *p* values between 0.001 and 0.01, as ‘very highly significant’ (***) with *p* values between 0.0001 and 0.001 and as ‘extremely significant’ (****) with p-values < 0.0001. Pairwise Pearson-correlation with t-test was used for correlation analyses. Statistical analyses and creation of diagrams were performed using Microsoft Excel 2016, GraphPad Prism, version 8.4.0 and 8.4.3 and R programming language, version 4.1.0.

## Results

In this project, TEM, fNTA-based and MBFCM EV detection technologies were used to characterize and quantify EVs in serum of leukemia and healthy samples, aiming to establish a proof-of-concept workflow to evaluate EVs as potential diagnostic/prognostic or predictive markers for clinical entities and to identify EV protein markers potentially predicting immune reactions in the serum leukemic compared to healthy samples. Enriched EVs derived from leukemia patients and healthy donors were first characterized by TEM and fNTA, and in a next step MBFCM was adapted to analyze EV surface protein compositions in minimally processed serum samples derived from leukemia patients versus healthy donors.

### Characterization of serum-derived EVs in leukemic and healthy control samples

**Fig. 1:**
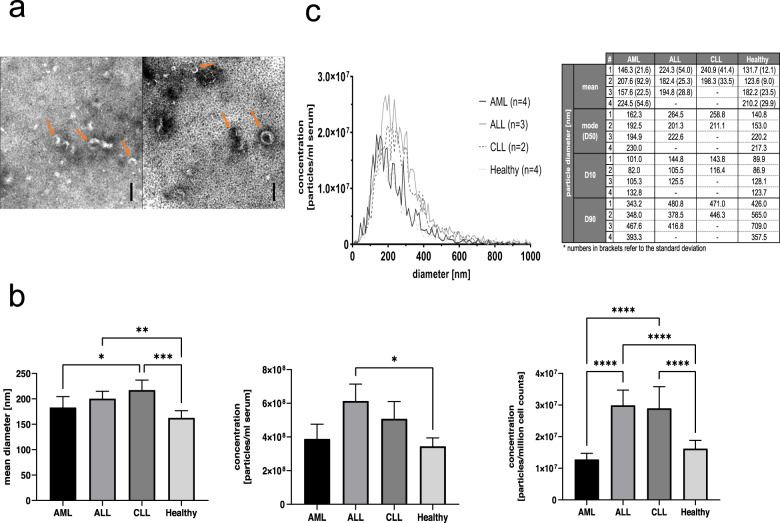
Identification and quantification of EVs purified from serum samples using TEM and fNTA. EVs were prepared by immunoaffinity enrichment applying Exosome isolation kit pan prior to an additional centrifugation. **a** TEM identifies EVs with typical cup-shaped appearance in healthy (left side) and AML (right side) serum samples. Scale bars are the same for all images and represent 100 nm. Arrows exemplarily highlight vesicular structures. **b** fNTA enables size and concentration analyses of EVs. Results of mean diameter (nm) and concentration measurements (particles/ml serum and particles/million cell counts) of purified EVs from AML (*n* = 4), ALL (*n* = 3), CLL (*n* = 2) and Healthy (*n* = 4) samples are given. Detected particle concentrations were corrected for sample dilution and normalized to serum cell counts. Data is given as mean +/− 95% CI of values. c Size distributions of EVs measured by fNTA are given in histograms and tables. #Orders with increasing patient numbers are given. Statistical analysis was done with GraphPad Prism, version 8.4.3, by one-way analysis of variance (ANOVA) with Benjamini-Hochberg adjustments for multiple comparisons. Differences were considered as ‘significant’ (*) with adjusted p-values between 0.01 and 0.05, as ‘highly significant’ (**) with adjusted *p* values between 0.001 and 0.01, as ‘very highly significant’ (***) with adjusted p-values between 0.0001 and 0.001 and as ‘extremely significant’ (****) with adjusted *p* values < 0.0001.

First, we performed TEM imaging, as recommended by the MISEV2018 guidelines, to characterize morphology of freshly prepared serum EVs and confirm successful enrichment thereof by an immunoaffinity-based strategy. Indeed, we could ascertain a typical cup-shaped appearance of serum EVs with heterogeneous desiccated diameters around 100 nm in both AML and healthy samples. No differences between EVs derived from patients versus healthy donors could be detected (Fig. 1a).

Next, we performed fNTA to quantify EV concentrations (particles/ml serum and particles/million cell counts) and assess size distribution profiles of purified EV samples from the peripheral blood of healthy donors and EVs derived from AML, ALL, and CLL patients (Fig. 1b, c). We utilized a protocol we previously optimized [21] and analyzed EVs derived from four AML-patients, three ALL-patients, two CLL-patients and four healthy donors. While conventional NTA only allows detection of total particles including non-EV particles, we here applied a protocol based on utilizing a fluorescent membrane dye to stain and quantify concentrations of stained EVs and not non-EV particles which was previously optimized by Mussack et al. [21]. Overall, mean diameters of particles ranged between 163 and 218 nm, as typically observed in EVs obtained after preparation by immunomagnetic separation. EVs obtained from healthy serum appeared with a (very) highly significantly lower diameter of 163 (95% CI: 149–177) nm compared to EVs derived from ALL- or CLL- serum with 201 (95% CI: 186–215) nm and 218 (95% CI: 198–237) nm, respectively (Fig. 1b). The diameter of CLL-derived EVs was even significantly larger compared to AML-derived EVs representing a mean diameter of 183 (95% CI: 162–205) nm (Fig. 1b).

In total, fNTA revealed average concentrations of 1.63 (95% CI: 1.37–1.88) x 10^7^ particles/million cell counts for healthy controls, 1.28 (95% CI: 1.09–1.47) x 10^7^ particles/million cell counts for AML patients, 2.99 (95% CI: 2.51–3.47) x 10^7^ particles/million cell counts for ALL patients, and 2.89 (95% CI: 2.21–3.58) x 10^7^ particles/million cell counts for CLL patients (Fig. 1b). The obtained normalized EV concentrations of ALL and CLL samples were extremely significantly higher compared to normalized EV concentrations of healthy and AML samples. Size distributions of EVs from healthy and leukemic sera appeared comparably with one peaking area around 200 nm (Fig. 1c).

### Robust characterization of EV surface protein signatures with MBFCM

Next, we performed MBFCM analyses to compare the EV surface protein expression on healthy donor versus leukemia patient-derived EVs. We previously have optimized an MBFCM-based assay for analysis of cell culture-derived EV and demonstrated that this assay also facilitates the detection of EV surface markers in different biological fluid samples [15]. Of note, we also showed that freeze thaw cycles do not affect detected EV surface marker profiles notably.

The MBFCM assay used here is based on the co-detection of two EV surface markers: One marker based on specificity of one of 37 capture beads coated with specific capture antibodies included in the assay, and the other marker based on the fluorescence-labelled detection antibody added, here a mixture of pan anti-tetraspanin (CD9, CD63, CD81) antibodies aiming to detect all tetraspanin-positive EVs bound to the respective capture bead [14]. The assay principle ensures that only EVs and not free proteins are detected, thereby facilitating specific detection of EVs without further purification, in both cell culture supernatants and biological fluids [15]. Detected signal quantities directly correlate with the abundance of respective surface proteins in EV samples. Analysis of serum samples with this MBFCM assay can lead to background or unspecific signals if EVs are not further purified, e.g. by size exclusion chromatography [15], which is why we previously have purified EVs before analysis, and normalized the assay input between different donors based on measured NTA particle concentrations. Since this doesn’t allow direct comparison of the abundance of respective EV surface proteins between donors and potentially can introduce a bias from purification steps, we here aimed to directly measure unprocessed serum samples instead.

**Fig. 2:**
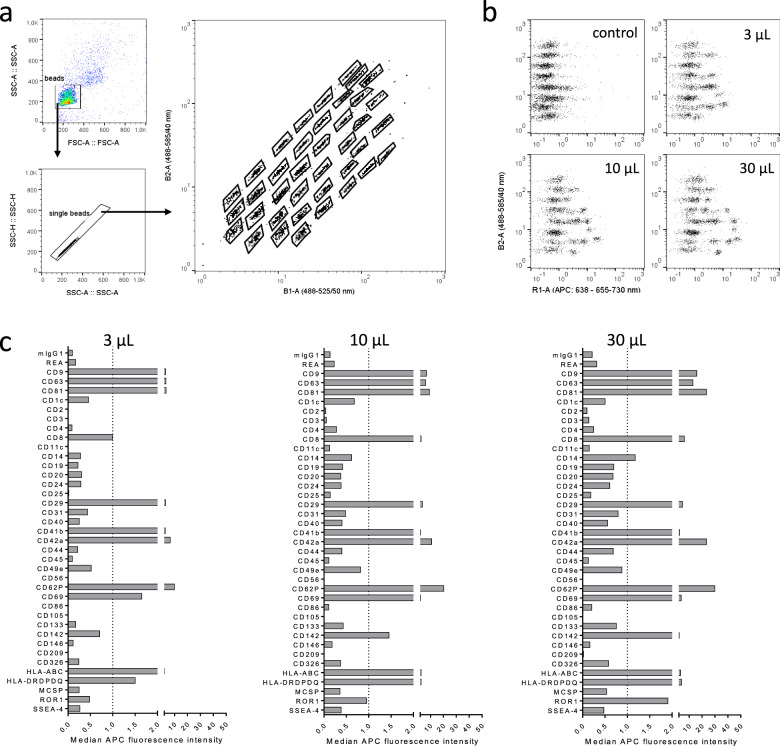
Establishment of a robust workflow to directly quantify EV surface protein expression in serumsamples by MBFCM. **a** Gating strategy applied to identify capture bead populations. **b** Examples for signals detected when using different seruminput volumes for the MBFCM assay. Control indicates procedural control without EVs but stained with pan-tetraspanin detection antibodies, volumes given indicate seruminput volumes. Allsamples were diluted in MACSPlex buffer to a final volume of 60 µL during the initial capture step. **c** Example data showing detected background-subtracted signal intensities for each marker at different seruminput volumes.

In a first step, we therefore measured blood serum samples at different input doses by MBFCM to establish a simple yet robust assay workflow suitable for relating data directly to abundance per blood volume without further sample processing. Capture beads were identified as described previously [15] (Fig. 2a). We chose serum input amounts of 3, 10, 30, and 60 µL in a total volume of 60 µL during the capture step (Fig. 2b). During processing, samples with 60 µL input regularly clogged the filter plates used which resulted in low bead counts and highly variable data (not shown). 3–30 µL input resulted in low background based on internal mIgG and REA isotype control signals, with highest signals detected for positive markers at 30 µL assay input (Fig. 2c). Based on these results, we decided to use 30 µL serum as assay input for pre-cleared and otherwise unprocessed serum samples throughout this study.

### MBFCM measurement of EV surface protein profiles on leukemia patient and healthy donor-derived serum samples

**Fig. 3:**
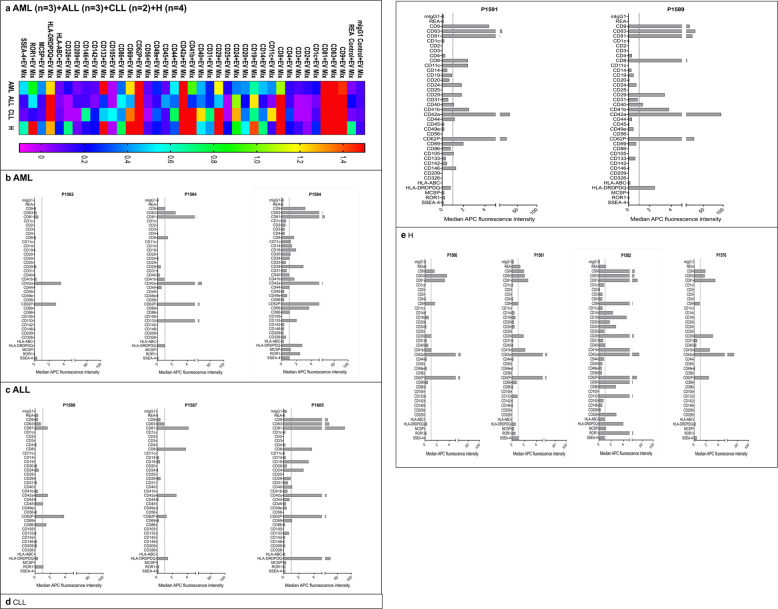
Identification of serum-derived EVs from AML, ALL, CLL and healthy samples by MBFCM. MBFCM allows the detection of EVs (co-) expressing 37 different antigens in a semi-quantitative manner. Results (median fluorescence intensity) obtained with AML (*n* = 3), ALL (*n* = 3), CLL (*n* = 2) and Healthy (H) (*n* = 4) samples are given in a heatmap analysis (Fig. 3 **a**). Data from single cases referring to individual P-numbers are given (Fig. 3 **b**–**e**). Median APC fluorescence intensities are displayed resulting from MACSPlex analysis of EVs isolated from serum of all samples.

In this study we included samples from patients diagnosed with AML (*n* = 3), ALL (*n* = 3), CLL (*n* = 2) and Healthy donor-derived samples (*n* = 4; Tables 1,2) and analyzed the EV surface protein profile by MBFCM. This assay comprises 39 hard-dyed capture bead populations (4 μm diameter), each of them coated with different monoclonal antibodies against 37 potential EV surface antigens or two internal isotype negative controls (details summarized in Table 3 [24–27]). Surface proteins included in the MBFCM assay comprise the tetraspanins CD9, CD63, and CD81, and other surface proteins such as various leukocyte, T cell (CD4, CD8), B cell (CD19, CD20, CD24), monocyte (CD14), thrombocyte (CD41b, CD42a, CD62Pa, CD69), integrin (CD11c (integrin αX or CR4), CD29 (integrin β1), CD41b (integrin αIIβ), CD49e (integrin α5)), endothelial (CD31, CD105, CD146 (Mel-CAM)), or MHC-associated (HLA-ABC (MHC-I), HLA-DRDPDQ (MHC-II)) associated antigens. MBFCM results obtained are given in Fig. 3. The commonly used EV markers CD9, CD63, and CD81 were detected on EVs in all measured samples, as expected (Fig. 3a). In addition, lineage-associated markers (e.g. CD8, CD42a, CD62P and HLA-DRDPDQ) were found on EVs in high expression in all sample entities, whereas the markers CD2, CD3, CD25, CD56, CD142, and CD209 showed low expression on EVs as given in the heatmap analysis. A more detailed presentation of results of single cases (Fig. 3b–e) showed that the thrombocyte or myeloid blast cell-associated markers (CD42a, CD62P, and CD133) were highly expressed on AML-sample-derived EVs (Fig. 3b), whereas T cell (CD8), thrombocyte (CD42a, CD62P) or MHC associated (HLA-DRDPDQ) markers were highly expressed on lymphoid leukemia-sample-derived EVs (Fig. 3c, d).

**Table 3:** List of antibodies used as capture antibodies bound to the polystyrene particles in the multiplex platform [24–27].

Capture antibody	Clone	Target
mIgG1	IS5-21F5	Isotype control
REA	REA293	Isotype control
CD9	SN4	Extracellular Vesicle marker
CD63	H5C6	Extracellular Vesicle marker
CD81	5A6	Extracellular Vesicle marker
CD1c	AD5-8E7	BDCA-1, major subpopulation of human myeloid dendritic cells
CD2	LT2.2	T cells, subset of NK cells
CD3	BW 264/56	mature human T cells, thymocytes, subset of NK cells
CD4	Vit-4.3	T helper cells, thymocytes, monocytes, dendritic cells
CD8	BW 135/80	cytotoxic T cells, thymocytes, subset of NK cells
CD11c	MJ4-27G12	integrin αX or CR4, monocytes, macrophages, NK cells, granulocytes, myeloid dendritic cells (MDCs), subsets of T and B cells
CD14	TÜK4	monocytes and macrophages, subset of neutrophils and myeloid dendritic cells
CD19	LT-19	B cells
CD20	LT20.34	B lineage cells from the pre-B cell stage to the B cell lymphoblast stage
CD24	32D12	heat-stable antigen (HSA)
CD25	3G10	activated T and B cells, macrophages, subset of non-activated CD4+ regulatory T cells
CD29	TS2/16.2.1	integrin beta 1
CD31	AC128	monocytes, platelets, and granulocytes
CD40	HB14	B cells, macrophages, dendritic cells, endothelial cells, fibroblasts, plasma cells, subset of peripheral T cells
CD41b	REA336	β chain of Integrin α-IIb, megakaryocytes, platelets
CD42a	REA209	Platelets, megakaryocytes
CD44	DB105	Cancer stem cells (CSC), hematopoietic, fibroblastic, and glial cells.
CD45	5B1	leukocyte common antigen
CD49e	NKI-SAM1	integrin α5 chain, lymphocytes, monocytes, fibroblasts, endothelial cells
CD56	REA196	neural cell adhesion molecule (NCAM), resting and activated NK cells, minor subset of CD3+ T cells
CD62P	REA389	P-selectin, vascular endothelial cells and platelets
CD69	FN50	Activated lymphocytes, monocytes, and platelets
CD86	FM95	B7-2, activated B and T cells, dendritic cells, and monocytes/macrophages
CD105	43A4E1.71	mature endothelial cells, some leukemic cells of B lymphoid and myeloid origin
CD133	AC133.1.6. 2.1.1	multipotent progenitor cells, including immature hematopoietic stem and progenitor cells, circulating endothelial progenitor cells, fetal neural stem cells, other tissue-specific stem cells, cancer stem cells
CD142	HTF-1	Tissue factor, activated endothelial cells, monocytes, macrophages, platelets, and some tumor cell types
CD146	541-10B2	MUC18, MCAM, Mel-CAM, endothelial cells, pericytes, smooth muscle cells, follicular dendritic cells, melanoma cells, sub-population of activated T lymphocytes, marrow stromal cells (MSCs)
CD209	DCN-47.5.4	DC-SIGN, dendritic cells, endothelial cells, macrophages, spleen
CD326	HEA125	EpCAM, basolateral surface of carcinoma and epithelial cells in tissues, circulating tumor cells, cancer stem cells, not on melanoma, neuroblastoma, sarcoma, lymphoma, leukemia cells, or normal fibroblasts
HLA-ABC	REA230	Nuclear cells
HLADP/DQ/D R	REA332	Antigen-presenting cells
MCSP	EP-1	melanoma-associated chondroitin sulfate proteoglycan antigen, melanoma tissues and melanoma cell lines but not carcinoma cells, fibroblastoid cells, and cells of hematopoietic origin
ROR1	2A2	receptor tyrosine kinase-like orphan receptor 1, chronic lymphocytic leukemia (CLL) and mantle cell lymphoma (McLellan), ovarian cancer, renal cancer, melanoma, and lung adenocarcinoma, adipose tissue, at early stages of B cell development
SSEA-4		Stage-specific embryonic antigen 4, undifferentiated human embryonic stem (ES) cells, induced pluripotent (iPS) cells embryonal carcinoma (EC) cells, and embryonic germ (EG) cells, somatic stem cells

On a first glance EVs from healthy samples also showed a high expression of CD8, CD19, CD29, CD41b, CD42a, CD62P, CD69 and ROR1 markers (Fig. 3e). This means that coexpression of several lineage-associated markers can be demonstrated in varying expressions in individual samples from leukemic and healthy sample donors.

### Differential serum-derived EV surface marker detection from leukemic and healthy samples as sorted by surface markers

**Fig. 4:**
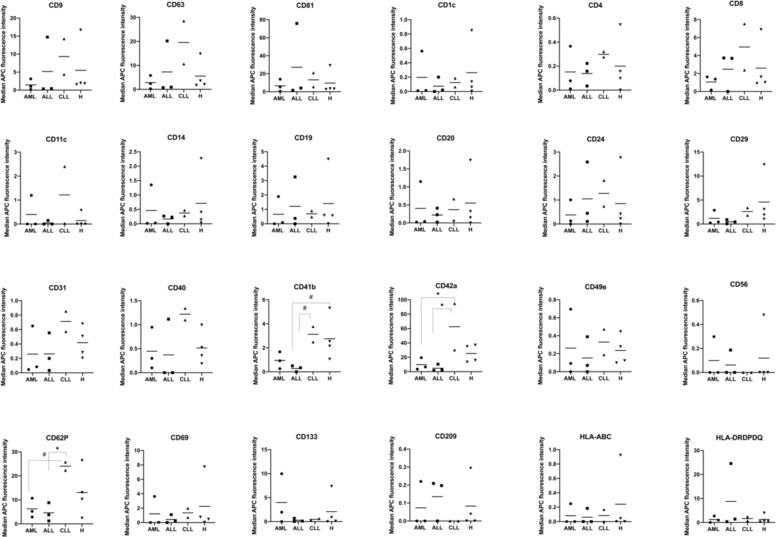
Quantification and comparison of EV surface marker expression in leukemic and healthy serum samples with MBFCM. Serum samples were analyzed by MBFCM. Differences in marker expressions on serum-derived EVs from AML (*n* = 3), ALL (*n* = 3), CLL (*n* = 2) and Healthy samples (*n* = 4) are shown in mean values. For statistical comparison of more than two groups one-way analysis of variance (ANOVA) with Benjamini-Hochberg adjustments for multiple comparisons was applied. Differences were considered as ‘borderline significant’ (^#^) with p-values between 0.05 and 0.1 and as ‘significant’ (*) with p-values between 0.01 and 0.05.

When sorting our results according to expressions of CD markers on EVs in different leukemic compared to healthy samples, we found remarkable differences (Fig. 4). Myeloid leukemic marker CD133 was detected with highest signal intensities in serum-derived EVs from AML compared to ALL, CLL, and Healthy samples. We further observed strong variations in signal intensities for CD209, which was not detected in CLL samples, and HLA-DRDPDQ, which showed the highest detection signals on serum-derived EVs from ALL samples. Of note, the expression of CD8, CD11c, CD31, CD40, CD41b, CD42a, and CD62P was detected at high signal intensities especially on serum-derived EVs from CLL samples. We observed a complete lack of signal detection for CD56 and CD209 on serum-derived EVs from CLL samples, while there was a clear signal on serum-derived EVs from AML, ALL, and Healthy samples (Fig. 4).

Significantly lower (*) signal intensities of CD42a positive serum-derived EVs were found in AML and ALL compared to CLL samples. Borderline significantly lower (^#^) signal intensities of CD62P positive serum-derived EVs were found in AML compared to CLL samples, while significantly lower (*) signal intensities of CD62P positive serum-derived EVs were found in ALL compared to CLL samples. Borderline significantly lower (^#^) signal intensities of CD41b positive serum-derived EVs were found in ALL compared to CLL and Healthy samples (Fig. 4).

### Differential EV-marker detection in leukemic or healthy samples normalized to several subgroups

In order to evaluate differences between leukemic and healthy samples in more detail, we normalized data of leukemic samples to healthy samples (a), to leukemic cell counts (b) and to WBC counts (c).

#### Differential EV-marker detection in leukemic samples normalized to healthy samples

**Fig. 5:**
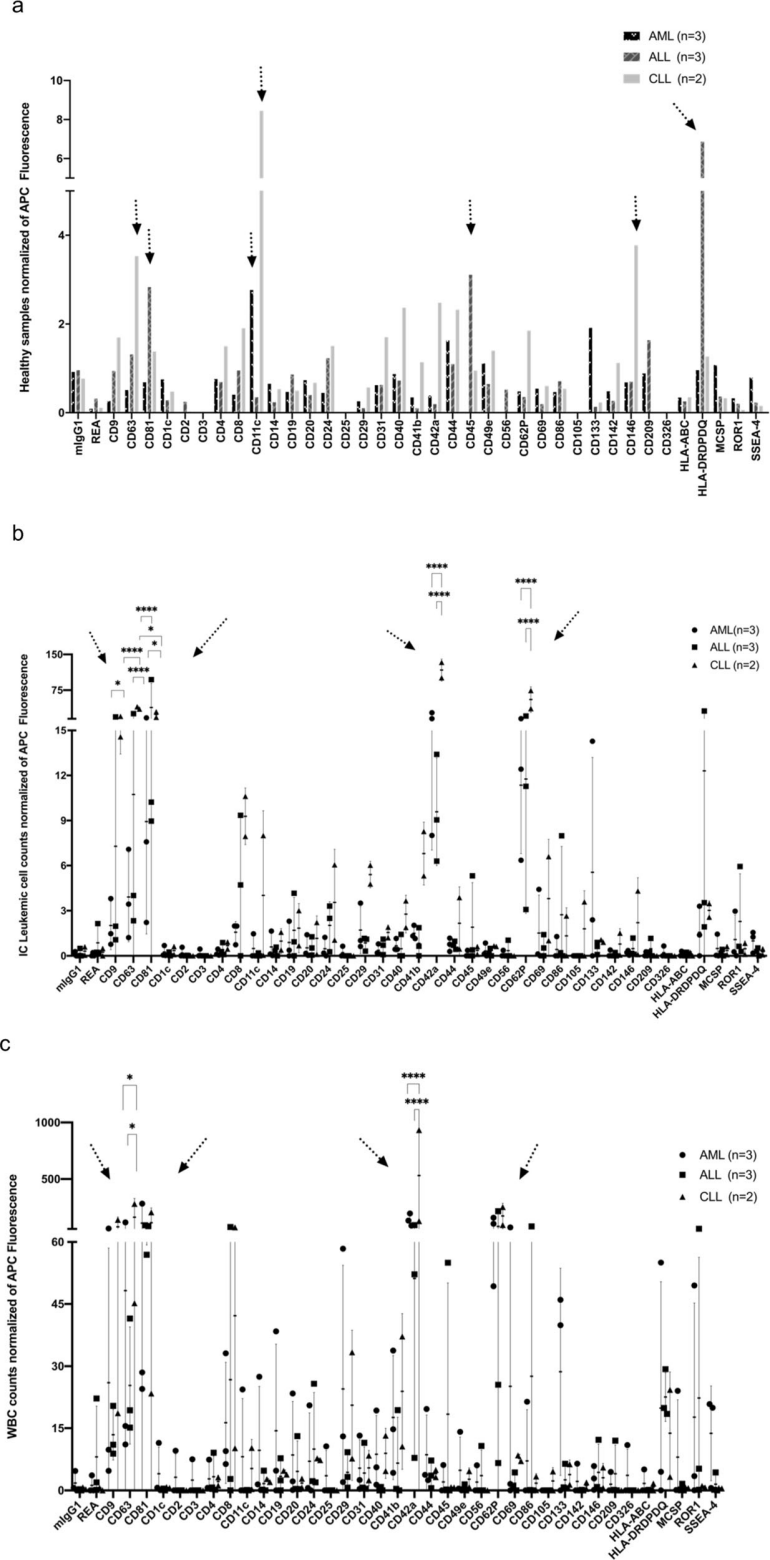
Identification and comparison of serum-derived EVs from leukemic after normalization to healthy data (**a**) IC leukemic cell counts (**b**) or WBC (**c**). MBFCM allows a semi-quantification and comparison of results in different diagnostic entities based on their (differential) expression profile of 37 different antigens. Results (median fluorescence intensities) normalized to healthy data (**a**), IC leukemic cell counts (**b**) or WBC-counts (**c**) are given. **a** Presentation of EV data normalized to healthy samples; **b** Presentation of EV data normalized to IC leukemic cell proportions; c Presentation of EV data normalized to WBC. **b**, **c** are given mean data with SD in individual dot plots. Fold changes (FC) of APC fluorescence of different marker expressions are given. Arrows point to the most abundant findings. For statistical comparison of more than two groups were analyzed applying one-way analysis of variance (ANOVA) with Benjamini-Hochberg adjustments for multiple comparisons. Differences were considered as ‘significant’ (*) with p-values between 0.01 and 0.05 and as ‘extremely significant’ (****) with p-values < 0.0001.

To detect and describe the differences between leukemic and healthy EV profiles in more detail we normalized results of leukemic samples to results obtained from healthy samples. As given in Fig. 5a we found higher fold-changes (> 1.5) of CD11c, CD44, CD133, and lower fold-changes (> 1) of CD49e and MCSP positive serum-derived EVs in AML than in healthy samples. We found higher fold-changes of CD81, CD45, HLA-DRDPDQ positive serum-derived EVs in ALL than in healthy samples. We found higher fold-changes of CD63, CD8, CD11c, CD40, CD42a, CD44, CD62P, and CD146 positive serum-derived EVs in CLL compared to healthy samples (Fig. 5a).

In summary, these data show that serum-derived EVs from AML, ALL, or CLL can be differentiated from healthy serum-derived EVs. Especially, AML derived EVs (positive for CD11c and CD133), ALL derived EVs (positive for CD45, HLA-DRDPDQ) and CLL derived EVs (positive for CD11c and CD146) were found in higher fold-changes compared to EVs derived from healthy samples.

#### Differential EV-marker detection in leukemic samples normalized to IC leukemic cells

In addition, we normalized results obtained from leukemia samples to IC leukemic cell proportions. As given in Fig. 5b, IC leukemic cell normalized signal intensities were higher for CD133 in AML than ALL and CLL. We found significantly lower signal intensities of CD81 (****) positive serum-derived EVs comparing AML with ALL derived EVs and significantly lower signal intensities of CD9 (*), of CD63 (****), of CD81 (*), of CD42a (****), of CD62P (****) positive serum-derived EVs comparing AML with CLL derived EVs with results normalized to IC leukemic cell counts.

We found significantly lower (****) signal intensities of CD63, CD42a, CD62P positive serum-derived EVs, but significantly higher (*) signal intensities of CD81 positive serum-derived EVs comparing ALL with CLL derived EVs with results normalized to IC leukemic cell counts (Fig. 5b).

#### Differential EV-marker detection in leukemic samples normalized to WBC counts

To detect and describe differences in more detail, we normalized MBFCM results of leukemic samples to WBC counts. As given in Fig. 5c, we found high signal intensities (positivity) of CD29, CD69, and CD133 on serum-derived EVs in AML when normalized to WBC counts.

We found high signal intensities (positivity) of CD86 and HLA-DRDPDQ on serum-derived EVs in ALL and high signal intensities of CD8, CD42a on serum-derived EVs in CLL when normalized to WBC counts. We found significantly lower signal intensities of CD63 (*), of CD42a (****) positive serum-derived EVs comparing AML with CLL derived EVs and significantly lower signal intensities of CD63 (*), of CD42a (****) positive serum-derived EVs comparing ALL with CLL derived EVs when normalized to WBC counts (Fig. 5c).

### Comparisons and correlation analyses of serum-derived EV marker expression by MBFCM and cellular marker expression by flow cytometry in leukemic and healthy samples

Possible relationships between EV marker expressions (as detected by MBFCM) and cellular marker expressions (as detected by cellular flow cytometry) in leukemic and healthy samples were assessed.

#### Correlation analyses of serum-derived EV and cellular marker expressions (monocytes, B cells, T cells) in leukemic and healthy samples

Since leukemic cells in none of the AML-cases were positive for CD14, we compared CD14 marker expressions on EVs from all pooled AML, ALL and healthy samples. We found a significant positive correlation between serum-derived EV and cellular positive CD14 marker expressions in pooled AML and ALL samples (*r* = 0.63, *p* = 0.04, *n* = 6), while a significant negative correlation of this marker was seen in healthy samples (*r* = -0.68, *p* = 0.05, *n* = 4). Moreover, we found a high negative correlation between serum-derived EV and cellular CD19 marker expressions in pooled AML and Healthy samples (*r* = -0.70, *p* = 0.34, *n* = 7). While a significant positive correlation between serum-derived EV and cellular CD19 marker expression was found in pooled ALL and CLL samples (*r* = 0.61, *p* = 0.0026, *n* = 5). Actually, we found a very weak negative correlation between the serum-derived EV and cellular CD3 marker expressions in pooled leukemic and healthy samples (CD3: *r* = −0.23, *p* = 0.0008, *n* = 11), and no correlation between the serum-derived EV and cellular CD56 marker expression in all leukemic and healthy samples was seen (CD56: *r* = 0.06, *p* = 0.0005, *n* = 12) (Supplementary Fig. 1).

#### Correlation analyses of serum-derived EV marker expressions with IC leukemic cells

We correlated leukemic cell counts (as evaluated by flow cytometry as ‘IC leukemic cells’ in leukemic samples) with leukemic cell-lineage-associated CD marker expressions on EVs (details are given in Tables 1,2).

There was a positive correlation between results obtained from serum-derived EV markers and results obtained from AML, ALL and CLL samples’ IC leukemic cells.

**Fig. 6:**
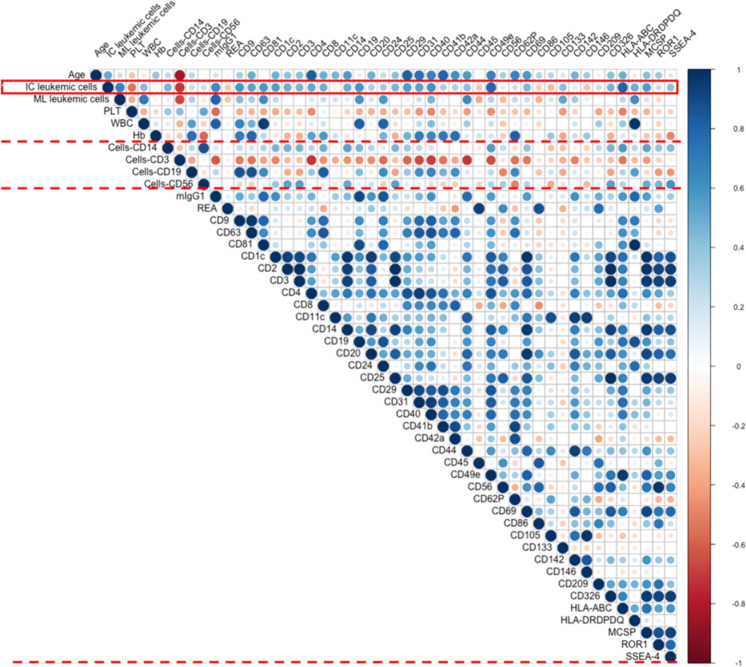
Correlation analyses of serum-derived EV markers (determined by MBFCM) with IC leukemic cells, cellular platelets (PLT) and white blood cells (WBC) in all leukemic samples. Correlation analyses of serum-derived EV marker expression (detected by MBFCM) with IC leukemic cells, cellular PLT and WBC in all AML, ALL, and CLL samples. Immune cytologically detected leukemic cells (IC leukemic cells), platelet counts (PLT) and white blood cells (WBC) are evaluated and given in Table 1. Especially correlation analyses of IC leukemic cells and serum-derived leukemic EV markers (CD19, CD133, CD24, CD44, CD142, MCSP, ROR1, SSEA-4), Integrin-related EV markers (CD11c, CD29, CD41b, CD49e), MHC-related EV-markers (HLA-ABC and HLA-DRDPDQ), platelet-related EV-markers (CD42a, CD62P, and CD69) and endothelial-related EV-markers (CD31and CD146), cellular PLT, WBC and serum-derived EV markers (CD41b, CD42a, CD62P, CD69, and CD40) were also supported. Blue color shows positive correlations (positive correlation coefficient r), red color shows negative correlations (negative correlation coefficient r). Statistical analyses and creation of diagrams were performed using Microsoft Excel 2016 and R programming language. H: healthy; r: Pearson’s correlation coefficient; n: numbers.

Moreover, we found a positive correlation between serum-derived EV CD24 (GPI-anchored protein) (*r* = 0.36, *p* = 0.3, *n* = 8), CD44 (*r* = 0.43, *p* = 0.2, *n* = 8), CD133 (*r* = 0.42, *p* = 0.2, *n* = 8), CD142 (*r* = 0.16, *p* = 0.6, *n* = 8), MCSP (*r* = 0.56, *p* = 0.1, *n* = 8), ROR1 (*r* = 0.23, *p* = 0.5, *n* = 8), SSEA-4 (*r* = 0.42, *p* = 0.3, *n* = 8) and IC leukemic cells in all pooled leukemic samples. A significant positive correlation between serum-derived EV leukemic cell marker CD19 (*r* = 0.68, *p* = 0.004, *n* = 5) and IC leukemic cells in ALL and CLL samples also was seen (Fig. 6).

A significant positive correlation was found between serum-derived EV CD29 (integrin β1) (*r* = 0.56, *p* = 0.1, *n* = 8), CD41b (integrin αIIβ) (*r* = 0.34, *p* = 0.4, *n* = 8), CD49e (integrin α5) (*r* = 0.80, *p* = 0.01, *n* = 8) and IC leukemic cells in AML, ALL and CLL samples. A low negative correlation between serum-derived EV CD11c (integrin αX or CR4) (*r* = -0.06, *p* = 0.8, *n* = 8) and IC leukemic cells (Fig. 6), and a significant positive correlation between serum-derived EV HLA-ABC (MHC-I) (*r* = 0.80, *p* = 0.01, *n* = 8) and HLA-DRDPDQ (MHC-II) expression (*r* = 0.49, *p* = 0.2, *n* = 8) and IC leukemic cells in AML, ALL, and CLL samples were found (Fig. 6).

Positive correlations were also observed between serum-derived platelet associated EV CD42a (*r* = 0.32, *p* = 0.4, *n* = 8) and CD62P (*r* = 0.23, *p* = 0.5, *n* = 8), CD69 (*r* = 0.45, *p* = 0.2, *n* = 8) with IC leukemic cells in AML, ALL and CLL samples (Fig. 6b), even though not significant.

There was a significant positive correlation between serum-derived endothelial associated EV CD31 (*r* = 0.54, *p* = 0.1, *n* = 8) and IC leukemic cells, while a negative correlation was seen between serum-derived endothelial associated EV CD146 (*r* = −0.29, *p* = 0.4, *n* = 8) and IC leukemic cells (Fig. 6).

#### Correlation analyses of serum-derived EV markers and cellular platelets (PLT), white blood cells (WBC) in AML, ALL, and CLL

We correlated PLT counts (as evaluated in leukemia samples Table 1) with thrombocytes-lineage-associated CD marker expressions on EVs.

We found a low and non-significant negative correlation between serum-derived platelet associated EV CD41b (integrin αIIβ) (*r* = -0.31, *p* = 0.4, *n* = 8), CD42a (*r* = −0,19, *p* = 0.6, *n* = 8), CD62P (*r* = −0.29, *p* = 0.4, *n* = 8), CD69 (*r* = −0.32, *p* = 0.4, *n* = 8) and platelets counts in all leukemic samples, while a positive correlation between serum-derived EV CD19 (*r* = 0.82, *p* = 0.01, *n* = 8), CD24 (*r* = 0.8, *p* = 0.01, *n* = 8), CD40 (*r* = 0.43, *p* = 0.2, *n* = 8) and cellular WBC in all leukemic samples were seen (Fig. 6).

In summary, the results presented in this study demonstrate that MBFCM facilitates assessment of EV surface protein compositions in biological fluid samples with minimal processing. Most importantly, this study also demonstrates that there is a correlation between serum-derived EV marker expression detected by MBFCM and cellular marker expression detected by flow cytometry or blood cell counting in leukemia and healthy samples. Especially a significant correlation on monocytes, B cells, T cells, blast markers, integrin associated markers, platelet associated markers, and endothelial associated marker could be assessed.

## Discussion

### Leukemia and prognosis

A focus of leukemia research lies on the development of new therapeutic strategies to reinduce an effective anti-leukemic immunity and the development of new diagnostic strategies to detect and monitor (risk associated) tumor-or immune-associated processes during the course of the disease [28].

### Monitoring of tumor load and immune reactions

Quantification and monitoring of leukemic cells (in peripheral blood (PB)) in chronic and acute leukemia is done by morphological, immunological (immunophenotyping) [29, 30], cytogenetic and molecular methods (e.g. PCR or FISH-analysis) [31]. New strategies with higher sensitivity like next-generation sequencing or digital droplet PCR expand the armamentarium for risk stratification, treatment monitoring and for the detection of minimal residual disease [32]. The advantage of these methods is their high sensitivity enabling the detection of low amounts of (mutated) DNA, including circulating tumor DNA also in blood samples- so-called liquid biopsies.

Despite these advantages, open questions are thresholds to differentiate malignant from benign mutated DNA or whether all relevant mutated gene sequences are addressed [32]. In addition, the full implementation of these novel diagnostic tools is expensive [33].

Quantification and qualitative monitoring of immune cells (especially in PB) is possible by refined immunophenotyping like multiparameter flow cytometry [29, 34].

### EVs: new particles of biologic significance

EVs are highly heterogeneous vesicles of different size originating from distinct subcellular compartments with a diverse molecular make up [35]. EVs carry a wide variety of proteins, including MHC molecules, chaperones, receptors, receptor ligands, cytokines, nucleic acids (i.e. mRNA, miRNA, DNA), and lipids [36]. The term ‘EV’ is used as an umbrella term for mainly microvesicles, exosomes, and apoptotic bodies. EVs are known to incorporate proteins, including cell type-specific markers from their parental cells and might therefore qualify to provide information to monitor malignant disease [37, 38]. Furthermore, it was shown that molecular profiles of secreted vesicles faithfully reproduce those of cancer cells [39]. It has been shown that EVs influence immune responses and tumor progression: on the one hand, EVs secreted by DCs have been shown to carry MHC-peptide complexes allowing efficient activation of T lymphocytes, thus displaying a potential as promoters of (adaptive) immune responses [11]. On the other hand suppressive effects, e.g. of leukemia EVs in a context of bone marrow-related stromal cells or hematopoietic progenitor cells have been demonstrated [10]. Up to now the influence of EVs produced by leukemic cell lines or prepared from plasma on immune reactive cells of several lines in ex vivo settings have been tested and ‘EV-derived suppressive or stimulatory effects’ to modulate immune reaction were deduced [12].

In vivo EVs delivered in experimental animals such as mice supported ex vivo data, that differentiation, expansion, migration to lymph nodes and survival of hematopoietic cells could be modulated by EVs [40].

### Potential of TEM and fNTA to detect and characterize EVs in leukemic and healthy serum

In the context of this project, EVs prepared from serum from healthy donors as well as from patients with leukemia should be analyzed and compared to potentially deduce strategies to monitor tumor- or immune-related EVs or processes.

Serum is known to contain mixtures of healthy (and malignant) cells and their EV- derivatives [12]. The ‘identity’ of captured healthy or leukemia-derived serum EVs in our settings was confirmed by TEM, although a differentiation of malignant and non-malignant EVs was not provided by TEM, demonstrating ‘cup shaped’ forms in both entities [18].

Performing fNTA from leukemic and healthy serum samples, we could detect differences in EV size and concentration: the mean diameters of particles in leukemic samples were higher than in healthy samples, the EV concentrations of ALL and CLL samples were significantly higher compared to healthy samples and EV concentration in AML samples were significantly lower compared to ALL and CLL samples.

This means that we can confirm that fNTA allows a ‘rough’ characterization, however no refined differentiation of leukemic compared to healthy EVs and especially no subclassification of tumor or immune derived EVs [15, 21].

### Multiplex bead-based flow cytometry assay for assessment of EV surface protein profiles on EVs derived from biological fluids

In general, it is meanwhile well known that the EV content, including the protein and surface marker composition, is probably strongly dependent on the cell source, the cells’ activation status, and multiple other parameters. Since any EV will show similar surface profiles than the cell type releasing them, analysis of EV surface signatures in the biological fluid has the potential to reveal any changes happening in abundance, frequency, and behavior of respective cell types releasing the EVs. Thus, it is a promising approach to identify EV surface marker profiles that correspond to certain diseases such as leukemia and ultimately use EV-based liquid biopsies for diagnosis and therapy decision making. Importantly, we here demonstrated that MBFCM can be used to detect and quantify EV surface proteins on EVs in blood serum samples without the need for any processing or enrichment steps. Blood samples were pre-cleared from cells and bigger aggregates merely based on centrifugation which is typical for biobanked samples. We titrated the input dose of serum volume and ultimately used 30 µL of human serum as input for MBFCM, diluted 1:1 with assay buffer. This has not led to increased assay background but still yielded clear signals for all samples included. Due to the specificity of this sandwich MBFCM assay and the requirement on both bead capture and binding of detection antibodies on the same EV in order to measure positive signals in this assay, non-EV contaminants like free protein, lipoproteins, and other molecules won’t be detected, thereby facilitating the direct analysis of minimally processed human serum samples without any purification steps. Thus, in contrast to an approach we used previously where assay input would be dosed based on NTA particle counts following sample isolation by SEC, the approach presented in this study facilitates the relation of measured signal intensities for respective markers directly to abundance in donor’s whole blood.

### MBFCM data presentation and correlation

MBFCM provides data for many different EV surface proteins, often resembling classical lineage-specific blood cell surface markers. In this proof-of-concept study, we evaluated different ways how to present MBFCM data and correlate the data with clinical parameters. In heatmap analyses and bar diagrams of our study, we demonstrated that we could detect EVs positive for CD9, CD63, and CD81 by MBFCM using 37 specific markers (Fig. 3), as already shown by Koliha et al. and Wiklander et al. [14, 15]. MBFCM was moreover shown to be sensitive enough in our hands to detect different EV surface markers in serum from leukemic and healthy samples, as already detected in other tumors or multiple cell type associated with EV-subclassed analyses by other groups [14, 24, 41, 42]. As shown in all entities and given in Fig. 3, lineage-associated EV markers CD8, CD42a, CD62P, and HLA-DRDPDQ were highly expressed in all sample entities. On a first glance and presenting data of single as well as of pooled cases, we found a high expression of some markers in leukemia or healthy serum samples: thrombocyte or myeloid leukemic cell-associated markers (CD42a, CD62P, and CD133) seemed to be highly expressed on AML sample derived EVs (Fig. 3b), T cell (CD8), thrombocyte (CD42a, CD62P) or MHC associated (HLA-DRDPDQ) markers seemed to be highly expressed on lymphoid leukemia-sample-derived EVs (Fig. 3c, d) and EVs from healthy samples also showed a high expression of CD8, CD19, CD29, CD41b, CD42a, CD62P, CD69, and ROR1 markers (Fig. 3e).

Focusing on certain EV markers and comparing their expression in different leukemic and healthy samples, we demonstrated differences: As given in Fig. 4, significantly lower frequencies of platelet-derived EV CD42a marker expression was found in AML and ALL compared to CLL samples, platelet-derived EV CD62P was found with (borderline significantly) lower frequencies in AML compared to CLL samples and significantly lower frequencies in ALL compared to CLL samples. Borderline significantly lower frequencies of platelet-derived EV CD41b were found in ALL compared to healthy samples. All platelet-derived EV markers (CD41b, CD42a, and CD62P) were expressed high in CLL. This could mean that the frequencies of these platelet EV markers are altered in different leukemic patients. Platelet microparticles have been also shown to be involved in metastasis, angiogenesis, and invasiveness in lung cancer [43], breast cancer [44], and melanoma [24]. Therefore, a surplus of platelet-derived EVs might indicate tumor progression.

In addition, analyses of plasma-derived EVs from healthy male athletes during the course of physical exhaustion showed elevated frequencies of platelet-derived markers CD41b, CD42a and CD62P positive on EVs, which could indicate that a release of EVs by activated platelets might not be restricted to large EVs (>500 nm), but also be related to smaller EV populations [42]. This could mean that platelet-derived EVs may play a role in exercise-triggered processes such as immune-modulation and inflammation-associated tissue regeneration even in healthy samples.

Interestingly, we found higher frequencies of EV-associated myeloid leukemic cell marker CD133 in AML. According to Tolba et al., cellular CD133 expression correlates with poor prognosis in AML patients [45]. Our findings might point to a positive relation of cellular CD133 with EV CD133 expression, although our patient samples were not tested for cellular CD133 expression.

In a next step, we tried to find relevant differences in EV frequencies when normalizing results from leukemic to healthy samples, IC leukemic cell counts, or WBC (Fig. 5). Compared to healthy samples we found elevated fold changes of CD133 in AML derived EVs, HLA-DRDPDQ in ALL derived EVs and of CD11c and CD146 in CLL derived EVs. As we discussed before, EV CD133 might have a closer relation with AML compared to ALL and CLL. Human leukocyte antigens (HLA) can be divided into HLA-A, B, and C which are encoded by major histocompatibility complex (MHC)-I and HLA-DP, DQ, and DR which are encoded by MHC-II. Here, we found HLA-ABC antigens expressed with low signal intensities on serum EVs in all samples. This might be due to a high percentage of EVs with low MHC-I expression, such as those secreted by NK cells and platelets. According to Merkenschlager et al., cellular MHC-II expression restrained growth of murine B-cell leukemia cell lines in vitro and in vi*vo*, independently of CD4+ T-cell surveillance [46]. Their results showed that MHC-II cells autonomously regulate the balance between self-renewal and differentiation of normal and malignant B cells. Our findings might point to a possible relation of MHC-II positive EVs with cellularly expressed MHC-II. Moreover, MHC-II positive EVs might also regulate differentiation of normal and malignant B cells.

An increased production of integrin CD11c (integrin αX or CR4) positive EVs was shown in melanoma patients [24]. We could also find an increased production of CD11c positive EVs in serum of CLL samples.

Umit et al. demonstrated that cellular CD11c is not only expressed on CLL including 259 CLL patients, but also on dendritic cells, macrophages and monocytes as a marker for inflammation [47]. In our context, this might reflect that released CD11c positive EVs and cellularly expressed CD11c could point to inflammation processes in CLL. Prolonged inflammation in the microenvironment of CLL cells may cause a pragmatically unfavorable susceptibility to autoimmune disorders and secondary tumors in CLL [47].

While platelet markers and lymphocytic markers are highly specific, markers like CD146 are regularly found on endothelial cells and on mesenchymal stromal cell subtypes and their derived EVs might point to an involvement of endothelial cells and mesenchymal stromal cells in these diseases [48].

Increased production and release of CD133 positive EVs to serum in AML, of HLA-DRDPDQ positive EVs to serum in ALL and of CD42a, CD62P positive EVs to serum in CLL were found after normalization of results to IC leukemic cell and WBC counts. This might reflect again that leukemic cell-derived EV marker CD133 and platelet-derived EV marker CD42a and CD62P have a clear correlation with the cellular expression of these markers [24, 45]. These studies confirm that EVs may play a role in immune modulation, and moreover, EVs liberated from AML, ALL, and CLL were hypothesized to be involved in multisystemic signaling mediating regeneration and long-term adaptive responses [14, 42].

### Comparison and correlation analysis between cellular and EV associated antigen expressions by MBFCM

Serum-derived EVs might provide information about the cell–cell interactions, resulting stimulations or inhibitions of immune cells. These correlations could be detected using MBFCM to quantify EVs and their corresponding cellular markers by flow cytometry in leukemic and healthy samples.

We found a significant positive correlation between serum-derived EV and cellular CD14 marker expressions in pooled AML and ALL samples. Although the detailed characteristics are not known, microvesicles isolated from plasma of advanced melanoma patients, but not from healthy donors were shown to address CD14+ monocytes, resulting in CD14+ suppressed T-cell functions (possible with downregulation of HLA-DR) [49]. These findings could suggest that an immunosuppressive circuit conduct (e.g. mediated by tumor cells) and the generation of suppressive myeloid cells through the release of circulating microvesicles might work without the need for cell-to-cell contact. Likewise, cellular CD14 is mainly associated with monocytes/macrophages, but also present on the surface of neutrophils, though at lower levels [50]. This could mean that CD14+ cells could produce EVs, that could mediate various reaction in leukemia and healthy samples.

We found a high negative correlation between serum-derived EV and cellular CD19 marker expression in AML and healthy samples, however, a significant positive correlation in pooled ALL and CLL samples. These findings might reflect different functions of CD19 positive EVs (in AML and healthy serum) compared to CD19 positive EVs (in ALL and CLL with CD19 being the leukemic cell marker).

According to Gutzeit et al., exosomes derived from Burkitt’s lymphoma cell lines induce B cell (CD19+ or CD20+) proliferation and differentiation towards a plasma cell-like phenotype with class-switched recombination [51]. Human B cell lymphomas produce EVs which carry components of the Wnt signaling pathway, transfer them to B cells, and thus promote tumor progression and stabilize the malignant phenotype [52]. We hypothesize that, along with the previously discussed elevated frequencies of CD19 positive EVs in ALL and CLL, down regulated frequencies of CD19 positive EVs from AML and healthy serum samples might indicate tumor progression and an attenuated immune response. Our findings might suggest that CD19 positive EVs derived from leukemia in general could induce proliferation and differentiation.

Additionally, a positive correlation for CD3 positive EVs and cellularly expressed CD3 was found, implying a contribution of T cells to the mixture of vesicles in serum, but with low correlation between EVs’ and cellular CD3 expression. It is known from the literature that T cell-derived EVs might transfer functional miRNAs to antigen-presenting cells (APCs) to generate an immune response [53]. This could mean that T cell-derived EVs might be able to support immune responses against tumors.

Studies of molecular EV profiles have indicated that there are significant differences in protein and nucleic acid content of EVs derived from tumor cells compared to that of EVs produced by normal cells [37].

Leukemia-derived EVs have been shown to suppress activities of various immune cells, to induce apoptosis of activated CD8 T cells, to promote the expansion of regulatory T cells (Treg), and to interfere with differentiation of DC, favoring the proliferation of myeloid-derived suppressor cells [54]. These attributes of tumor-derived EVs are a manifestation of their distinctive molecular profiles and seem to be general phenomena in cancer. Interestingly, many of the proteins (e.g. oncoproteins, oncogenes, transcripts of proteins) found in tumor-derived EVs are well-known for their role in promoting tumor progression. Many of these proteins are also involved in inflammatory reactions, chemokine receptors, immunosuppressive ligands, or soluble factors involved in regulating angiogenesis [55, 56]. This suggests that tumor-derived EVs might play a critical role in cancer development and progression, although much of the currently available evidence originates from in vitro studies. In vivo evidence for exosomes as drivers of cancer pathogenesis is still incomplete.

### Clinical correlation analyses between clinical diagnosis and EV expression by MBFCM

Correlations of experimental findings with clinical subtypes of leukemia could contribute to refine the classification of the disease. We found direct correlations between immune cytologically detected leukemic cells (IC leukemic cells) with EV marker expressions (e.g. of CD24 (GPI-anchored protein), CD44, CD133, CD142, MCSP, ROR1, SSEA-4) as evaluated by MBFCM.

Detection of lymphoid-derived EV marker CD19 or myeloid-derived EV marker CD133 in leukemia could contribute to refine detection of residual disease [24, 45].

Our data moreover might point to a role of integrins: EV-associated integrinmarkers such as CD11c (integrin αX or CR4), CD29 (integrin β-1), CD41b (integrin αIIβ) and CD49e (integrin α-5) as described here could play a role for targeting special EV subtypes. CD29 is known as an interaction partner of tetraspanins on cells and is probably transferred together with tetraspanins to exosomes during their biogenesis [24]. Accordingly, CD29 had already been detected on different types of exosomes [57]. CD49e (integrin α5) was considered as a potential marker for melanoma-derived EVs because it was found to be expressed by melanoma cells and we detected CD49e signals on EVs from melanoma cell cultures [24].

Our data might point to an additional role in leukemia: we found a positive correlation of EVs positive for CD29, CD41b and CD49e with IC leukemic cells in all pooled leukemia samples. These findings might suggest that the complexity of EV signaling in leukemia and expand the spectrum of conceivable functions of EVs.

In addition, platelet-derived EV markers CD42a, CD62P and CD69 showed direct positive correlations with IC leukemic cell counts. By contrast, a negative correlation of these markers with platelet counts was shown. These findings could confirm again that platelet microparticles might be involved in metastasis, angiogenesis and invasiveness, might indicate tumor progression [24, 42, 43].

We found a direct positive correlation of EV-associated markers HLA-ABC (MHC-I) and HLA-DRDPDQ (MHC-II) with IC leukemic cells in all leukemia samples. MHC-peptide complexes on exosomes can be presented to T cells either in a direct or an indirect manner [10, 13]. Zitvogel and coworkers demonstrated that murine bone marrow-derived DCs secreted EVs carrying MHC-I, MHC-II, and T cell costimulatory molecules, leading to a priming of tumor-specific cytotoxic T lymphocytes (CTLs) and suppress tumor growth in vivo [13, 16, 58]. In our context this could mean that HLA-ABC and HLA-DRDPDQ positive EVs might directly indicate progressive in leukemia.

LAMA84 CML cell-derived EVs are able to alter functions of various tissue cells, including endothelial cells (EC), and thus exert proangiogenic effects [59]. Similar effects on angiogenesis were induced by EVs from K562 cells [60]. Here we detected a positive correlation of EV marker CD31 and a negative correlation of EV marker CD146 with IC leukemic cells. Overall, these data emphasize that exosomes released from leukemic cells could directly affect EC and modulate the process of neovascularization.

## Conclusion

In summary, we have comprehensively evaluated and optimized MBFCM based EV detection technology: MBFCM can not only quantify robust EV surface signatures in a given sample but is also useful for comparing differentially expressed surface markers between samples. It thereby facilitates the identification of heterogeneities between different EV sources, which may lead to the identification of EV markers being specific for certain cell types.

Our own data and findings of the literatures suggest that EVs may play a role in immune modulation, inflammation-associated tissue regeneration and regulation of coagulation. Although it is not yet clear which signals trigger the release of EVs from all these cell types and the target cells.

EV profiling might qualify as a highly reliable strategy to indicate the involvement of different subtypes in leukemia or in the mediation of antitumor reaction in leukemia compared to healthy samples. MBFCM is qualified as a suitable marker to detect heterogeneity of EV markers as well as the role of specific EVs in the classification of diseases as well as the monitoring of (disease related or unrelated) of EVs derived and released from cells.

However, the results obtained from this assay could be influenced by several factors, including cross-linking of beads by single EVs binding to more than one bead population and thus should be interpreted not as a single vesicle quantification.

In general, the combination of this rather robust and fast approach with more dedicated methods to validate candidate surface markers distinguishing EV subpopulations (i.e., single EV flow cytometric analysis cell sorting or detection) would pave the way to studying the function of EV subsets, which will be of the highest relevance to further improve our understanding of their molecular content and related functions. In addition, new applications provide a potential prognostic role or might allow to monitor the disease under the influence of new (immune) therapeutic approaches.

## Supplementary information

Supplementary Fig. 1

## Data Availability

All data generated or analysed during this study are included in this published article [and its supplementary information files].
