# Supplementary material for: Description and optimization of a multiplex bead-based flow cytometry method (MBFCM) to characterize extracellular vesicles in serum samples from patients with hematological malignancies
Source: Cancer Gene Ther. 2022 Apr 27;29(11):1600–15. doi: 10.1038/s41417-022-00466-1 (PMC9663305; doi:10.1038/s41417-022-00466-1)

Supplementary Fig.1 Correlation analyses of serum-derived EV markers and cellular markers CD14, CD19, CD3 and CD56 in leukemic and healthy samples

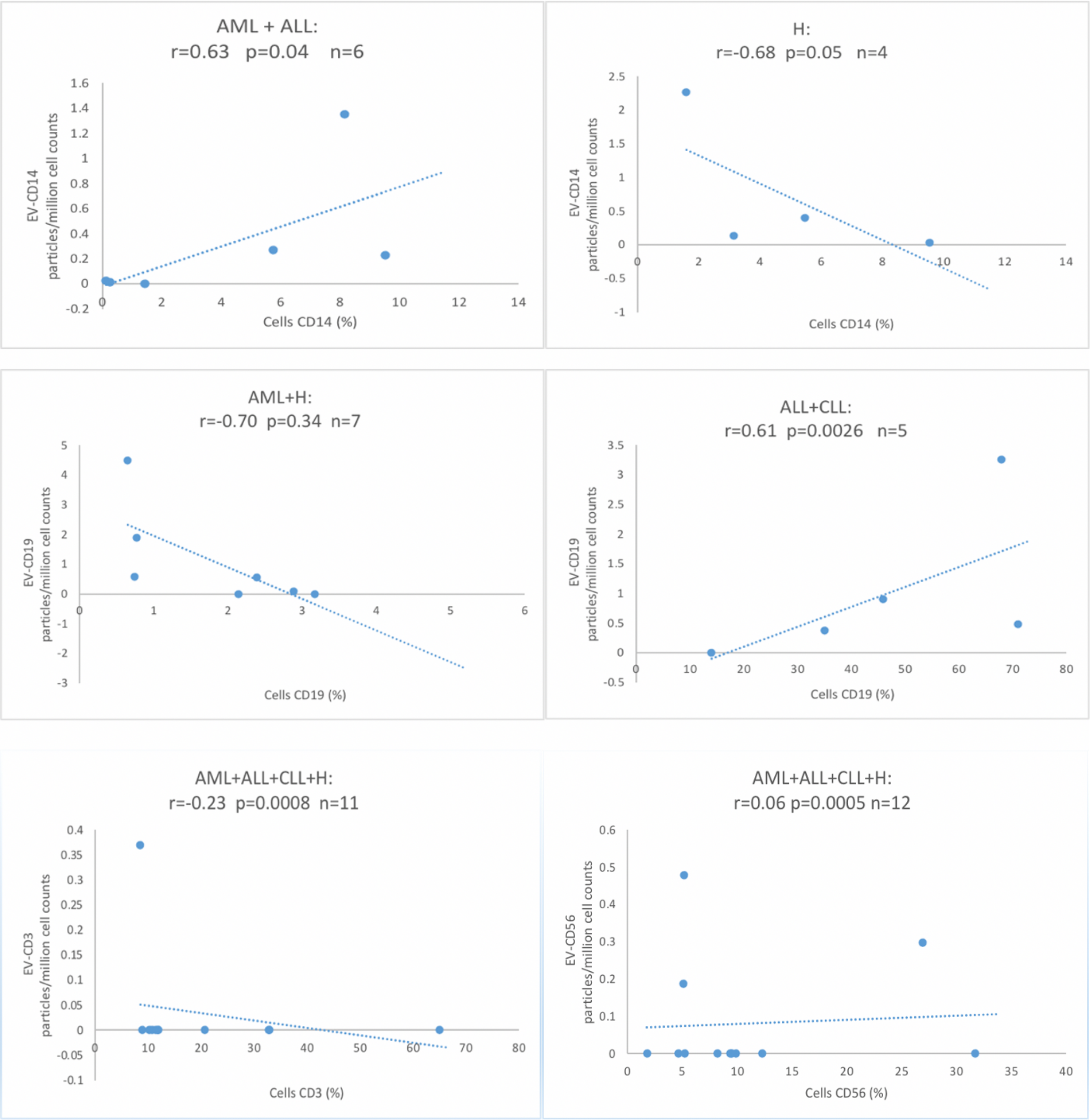

Supplement: Supplementary file 1 — Supplementary Fig. 1 [file 41417_2022_466_MOESM1_ESM.pdf]
